# Low-Dose Naltrexone for Managing Pain and Autonomic Symptoms in Patients With Dysautonomia

**DOI:** 10.7759/cureus.86538

**Published:** 2025-06-22

**Authors:** Nicolas Zapata, Emily Georgiadi, Christopher Cantrell, Ryan G Rilinger, Mackaleigh A Levine, Robert Wilson

**Affiliations:** 1 Department of Neurology, Cleveland Clinic, Cleveland, USA; 2 Cleveland Clinic Lerner College of Medicine, Cleveland Clinic, Cleveland, USA

**Keywords:** autonomic neurology, dysautonomia, low-dose naltrexone, pain treatment, postural orthostatic tachycardia syndrome (pots)

## Abstract

Introduction

Low-dose naltrexone (LDN) has been studied in recent years as a novel off-label therapy for several conditions under the umbrella of dysautonomia, which is defined as disorders affecting the autonomic nervous system (ANS), including postural orthostatic tachycardia syndrome (POTS). Naltrexone has a paradoxical pain-reducing effect in low doses due to transient opioid receptor blockage that increases compensatory endogenous opioid signaling. It is also thought that LDN may improve autonomic symptoms by reducing microglial activation via TLR-4 antagonism and subsequently counteracting central sensitization. Patients with dysautonomia often experience comorbidities such as small fiber neuropathy and fibromyalgia. The goal of this study was to gain a better understanding of LDN’s impact on autonomic symptoms and pain in patients with dysautonomia.

Methods

In this chart review, we analyzed the records of 29 patients diagnosed with dysautonomia (general, POTS, or stiff person syndrome). Information collected included demographics, comorbidities, reasons for LDN prescription, LDN dose (initial and final), documented pain changes, and Composite Autonomic Symptom Score-31 (COMPASS-31). COMPASS-31 is a validated questionnaire used to measure autonomic symptom burden. COMPASS-31 scores (including subsections) were collected from patients during their initial visit to our tertiary care autonomic center, the visit when LDN was prescribed, and a follow-up visit three to nine months later. Student’s t-test was used to determine statistical significance between COMPASS-31 scores from the initial visit and the LDN prescription visit, as well as between the LDN prescription visit and the follow-up visit.

Results

The most common reason for prescribing LDN to patients in this study was pain or fibromyalgia (61.11%), followed by orthostatic intolerance (27.78%). Improvement in pain was documented for seven patients (24.14%) at the follow-up visit after starting LDN. Most patients (86.21%) began LDN at a dose of 1 mg daily, but 11 subjects had an increased dose by their follow-up visit. No statistical significance was seen when comparing average COMPASS-31 total and subsection scores between the initial visit and the LDN prescription visit or between the LDN prescription visit and the follow-up visit. LDN therapy was largely tolerated with five patients reporting mild side effects.

Conclusion

LDN may be prescribed for patients with dysautonomia either due to autonomic dysfunction or pain. Patients might show improvement in pain within a matter of months, but the reason why some respond better than others remains unclear. Future studies are needed to understand how LDN can impact autonomic symptoms on an individual level. With further investigation, we might discover predictors of a strong therapeutic effect from LDN in patients with dysautonomia.

## Introduction

Dysautonomia encompasses a group of disorders marked by impaired regulation of autonomic functions such as heart rate, blood pressure, and temperature control [1,2]. Notable examples include postural orthostatic tachycardia syndrome (POTS) and fibromyalgia [3,4]. Patients with autonomic dysfunction may experience a wide range of symptoms, including palpitations, orthostatic intolerance, neck and shoulder pain, heat intolerance, and altered sweating patterns. Gastrointestinal disturbances, such as constipation, diarrhea, and delayed gastric emptying, are also common, alongside increased urinary frequency and incontinence. Fatigue and syncope frequently contribute to the chronic and often debilitating nature of these conditions. 

Current treatment strategies for dysautonomia are symptom-specific and may include beta blockers, midodrine, fludrocortisone, ivabradine, and pyridostigmine [2-4]. Recently, low-dose naltrexone (LDN) has been introduced in our tertiary care center’s autonomic clinic as a potential therapy for POTS, with some patients reporting improvements in symptom burden. Traditionally used at doses of 50-100 mg for treating alcohol and opioid dependence, naltrexone has also shown promise at significantly lower doses (up to 4.5 mg) for its anti-inflammatory and analgesic effects [5-7]. LDN’s transient blockade of opioid receptors may enhance endogenous opioid activity, thereby reducing pain [8,9]. Additionally, its antagonism of Toll-like receptor 4 (TLR-4) may inhibit microglial activation and cytokine signaling, potentially alleviating central sensitization and improving autonomic function [8,9]. TLR-4 inhibition may also reduce pro-inflammatory cytokines, potentially relieving gastrointestinal symptoms such as colitis. LDN has been explored as a treatment for fibromyalgia, neuropathy, and irritable bowel syndrome, with encouraging results in symptom reduction [5,10,11]. 

This study investigates the effects of LDN therapy on pain and autonomic symptom burden in patients with dysautonomia, using patient-reported outcomes. By evaluating LDN’s therapeutic potential, we aim to support its role in improving chronic pain management and orthostatic intolerance in this patient population. 

## Materials and methods

Chart review 

This IRB-approved retrospective chart review examined the electronic medical record (EPIC) of patients who received LDN therapy at our single tertiary autonomic care center. The inclusion criteria included patients evaluated by a neurologist at our tertiary center, those who had been on LDN therapy for at least three months, had a follow-up visit between three and nine months after LDN initiation, and had at least one recorded clinically distributed Composite Autonomic Symptom Score-31 (COMPASS-31) survey score. The exclusion criteria included patients evaluated by a neurologist outside our tertiary center, those not on LDN for at least three months, those who did not have a follow-up appointment three to nine months after LDN initiation, and those who did not have at least one recorded clinically distributed COMPASS-31 survey score.

The COMPASS-31 is a validated questionnaire used to quantify autonomic symptom burden, with higher scores indicating more severe symptoms [12]. The questionnaire is sent via the electronic medical record e-messaging system (MyChart) for clinical purposes before patient appointments. Total and subdomain scores were compared across three timepoints: initial visit, LDN prescription visit, and final follow-up visit.

A total of 29 patients who met these criteria were included in the analysis. For each patient, the following data were extracted: demographics (age, sex, race, BMI), relevant medical history (including chronic fatigue syndrome, fibromyalgia, mast cell activation syndrome, anxiety, depression, and small fiber neuropathy), LDN dosage (initial and final), and results from the COMPASS-31 autonomic symptom questionnaire. 

Pain outcomes were assessed based on self-reported pain levels documented in neurology clinic notes and categorized as follows: no change, improved, worsened, or not reported. Fatigue and brain fog were similarly evaluated using physician documentation. Only visits occurring within three to nine months before and after LDN initiation were included in the review. The clinical rationale for initiating LDN was also categorized based on documentation from the prescribing neurology visit.

Statistical analysis

All statistical analyses were conducted using R. Paired Student’s t-tests were used to assess changes in COMPASS-31 scores between timepoints. To account for multiple comparisons (n = 14), a Bonferroni correction was applied, resulting in a corrected significance threshold of α = 0.00357. 

## Results

The study included 29 patients with an average age of 50 years (range: 23-76). The majority were female (82.76%) and White participants (89.66%), with an average BMI of 27.71. The primary diagnosis was POTS in 62.07% of patients, followed by dysautonomia (34.48%) and stiff person syndrome (3.49%). Common comorbidities included anxiety (55.17%), small fiber neuropathy (51.72%), fibromyalgia (48.28%), and depression (48.28%). Chronic fatigue syndrome and mast cell activation syndrome were also present in 24.14% and 20.69% of patients, respectively (Table 1).

**Table 1:** Patient characteristics and comorbidities. POTS: postural orthostatic tachycardia syndrome.

Characteristic	Value, n=29
Age, mean (range)	50 (23–76)
Female, %	82.76
White, %	89.66
BMI, mean (SD)	27.71 (8.17)
Diagnosis	n=29
POTS	18 (62.07%)
Dysautonomia	10 (34.48%)
Stiff person syndrome	1 (3.49%)
Comorbidities	n=29
Anxiety	16 (55.17%)
Small fiber neuropathy	15 (51.72%)
Fibromyalgia	14 (48.28%)
Depression	14 (48.28%)
Chronic fatigue syndrome	7 (24.14%)
Mast cell activation syndrome	6 (20.69%)

The most common reason for initiating LDN therapy was pain or fibromyalgia (61.11%), followed by orthostatic intolerance (27.78%), neuronal excitability/gut motility (11.11%), and fatigue (11.11%). Regarding pain outcomes, 24.14% of patients reported improvement, 31.03% reported no change, 13.79% reported worsening, and 31.03% had no documentation regarding pain change (Table 2).

**Table 2:** Reason for starting LDN and pain outcomes. The n=18 refers to the number of patients represented in the table, while the numbers listed below (11, 5, 2, 2) represent the total number of times each particular reason was listed in a patient note. LDN: low-dose naltrexone.

Reason for starting LDN	n=18 (%)
Pain/fibromyalgia	11 (61.11)
Orthostatic intolerance	5 (27.78)
Neuronal excitability/gut motility	2 (11.11)
Fatigue	2 (11.11)
Change in pain with LDN	n=29 (%)
Improved	7 (24.14)
Same	9 (31.03)
Worsened	4 (13.79)
Not reported	9 (31.03)

Most patients (86.21%) began LDN at a dose of 1 mg. Final doses varied, with some patients titrating up to 4.5 mg. By the follow-up visit, 37.93% had increased their dose, and 58.62% remained at the same dose. Only one patient had their dose reduced, which was prompted by the patient's request to taper and discontinue LDN after not experiencing symptom improvement. LDN was generally well tolerated, with 17.24% of patients reporting side effects such as insomnia, nocturia, nausea, migraines, brain fog, and nightmares (Tables 3, 4). 

**Table 3:** LDN dosing. LDN: low-dose naltrexone.

Dose (mg)	Initial dose, n=29 (%)	Final dose, n=29(%)
0.5	1 (3.45)	0 (0)
1.0	25 (86.21)	16 (55.17)
1.5	0 (0)	1 (3.45)
2.0	2 (6.90)	5 (17.24)
3.0	1 (3.45)	4 (13.79)
4.0	0 (0)	2 (6.90)
4.5	0 (0)	1 (3.45)

**Table 4:** LDN tolerability. LDN: low-dose naltrexone.

Dose change	n=29 (%)
Increased	11 (37.93)
No change	17 (58.62)
Decreased	1 (3.45%)
Side effects	n=29 (%)
Reported	5 (17.24)

Eighteen patients completed the COMPASS-31 survey at the initial visit, 20 at the LDN prescription visit, and 15 at the final follow-up. One initial survey included only the total score and lacked subsection details (Table 4). The average total COMPASS-31 scores did not differ significantly between the initial visit (48.35, n=18) and the prescription visit (41.74; p=0.1334). Similarly, there was no significant difference in total scores between the prescription visit (41.74) and the final visit (48.53; p=0.2096). Statistical analysis also revealed no significant differences in any of the subsection scores between the initial and prescription visits or between the prescription and final visits (Table 5). 

**Table 5:** COMPASS-31 scores across timepoints. COMPASS: Composite Autonomic Symptom Score; LDN: low-dose naltrexone.

Score category	Initial visit (n=17)	LDN visit (n=20)	Final visit (n=15)	Initial–LDN p-value	LDN–final p-value
COMPASS-31 total score	48.35	41.74	48.53	0.1334	0.2096
Orthostatic intolerance	27.06	20.80	23.47	0.0239	0.3141
Vasomotor	2.00	1.82	2.30	0.7456	0.3936
Secretomotor	4.86	4.84	7.09	0.9842	0.0971
Gastrointestinal	8.74	9.51	10.31	0.6961	0.6940
Bladder	1.86	1.27	2.05	0.4228	0.2842
Pupillomotor	2.74	2.53	3.31	0.6495	0.0409

## Discussion

The primary objective of this study was to evaluate the changes in autonomic symptom burden among patients with POTS following initiation of LDN therapy. Additionally, we aimed to characterize this patient subgroup in terms of demographics, medical history, indications for LDN use, and prescribed dosages. Overall, LDN was well tolerated, with few reported side effects. However, as with any therapy, it is essential to consider individual patient perspectives on tolerability.

The demographic profile of our cohort was consistent with expectations for a dysautonomia population. The majority were female and White participants, aligning with findings from our previous studies on dysautonomia [13-15]. Notably, the average age in this study was 50 years, which is higher than in our prior POTS-focused cohorts (27-36 years) [13-15]. The prevalence of small fiber neuropathy and fibromyalgia as comorbidities also reflects previously reported indications for LDN, particularly in the context of chronic pain [6]. 

Pain and fibromyalgia were the most frequently cited reasons for initiating LDN therapy. LDN was started due to previous treatment failures. Given LDN’s proposed mechanism of reducing central sensitization via microglial inhibition, it may also offer benefits for orthostatic intolerance that is refractory to standard interventions [10,16,17]. A prior case study reported improvements in daily functioning and reductions in non-pain dysautonomia symptoms following LDN initiation [14]. Although fatigue and gastrointestinal dysmotility were less commonly cited as reasons for LDN use, even modest improvements in these debilitating symptoms could significantly enhance quality of life for dysautonomia patients [18]. 

In our study, COMPASS-31 scores did not show statistically significant changes across the three time points. While our initial intent was to track individual patient scores longitudinally, this was limited by incomplete survey data; only five patients had COMPASS-31 scores available at all three visits. As a result, analyses were based on group averages at each time point, reducing the ability to detect within-subject changes. These limitations are inherent to retrospective study designs and underscore the subjective nature of patient-reported outcomes. Although 24.14% of patients for whom pain outcomes were recorded reported improvement, the most common outcome was no change in pain. This variability in therapeutic response should be communicated to patients during shared decision-making to help set realistic expectations.

The wide range of LDN dosages prescribed in this study may have contributed to the variability in outcomes. Future dose-controlled studies could help clarify whether specific dosing regimens are more effective for managing symptoms of dysautonomia. Additionally, prospective studies that closely monitor symptom progression before and after LDN initiation would provide more robust evidence of efficacy.

Our findings suggest an inconsistent response to LDN among patients with dysautonomia, with some reporting benefit, others no change, and no statistically significant improvements in measured outcomes. It remains unclear which patient subgroups are most likely to respond favorably to LDN. Identifying demographic or clinical predictors of response could enhance clinical decision-making regarding LDN use. Placebo-controlled trials, with efforts to minimize confounding from concurrent therapies and comorbidities, are needed to better define the role of LDN in dysautonomia management. High-quality evidence from such trials will be essential to determine the ideal patient population and dosing strategy for maximizing therapeutic benefit. 

## Conclusions

This study found a varied response to LDN among patients with dysautonomia, with some reporting symptom or pain improvement, particularly in conditions like POTS and stiff person syndrome, while others did not see any benefit. Although group-level effects were not statistically significant, nearly a quarter of patients experienced pain relief, often requiring dose adjustments from the initial 1 mg. It remains unclear which patient subgroups are most likely to benefit from LDN. Although this study did not identify statistically significant changes in autonomic symptom burden, the findings underscore the need for randomized, controlled trials to identify predictors of response, optimize dosing, and control for confounding factors such as comorbidities and concurrent medications, ultimately guiding more personalized treatment strategies in this complex and heterogeneous patient population. 

.
